# Effectiveness of silver diamine fluoride versus sodium fluoride varnish combined with mother’s motivational interviewing for arresting early childhood caries: a randomized clinical trial

**DOI:** 10.1186/s12903-023-03456-3

**Published:** 2023-10-04

**Authors:** Randa Yassin, Hala Amer, Maha El Tantawi

**Affiliations:** Department of Pediatric Dentistry and Dental Public Health, Faculty of Dentistry, Alexandria University, Alexandria, Egypt

**Keywords:** Early childhood caries, Silver diamine fluoride, Sodium fluoride, Motivational interviewing, Caries arrest

## Abstract

**Background:**

Silver Diamine Fluoride (SDF) has gained attention as one of the minimally invasive modalities to manage ECC although it causes black staining of treated carious lesions. A possible affordable alternative may be Sodium Fluoride (NaF) varnish combined with good oral hygiene promoted by parental Motivational Interviewing (MI). The study compared the effectiveness of 38% SDF solution and 5% NaF varnish supported by parental MI in arresting ECC.

**Materials and methods:**

Children aged ≤ 4 years old with at least one active carious lesion (ICDAS score ≥ 3) were randomly assigned to treatment by a single application of 38% SDF solution or a single application of 5% NaF varnish supported by two MI sessions for mothers at baseline and after three months. Chi-Squared test was used to compare groups and multilevel logistic regression analysis was used to assess the effect of the interventions on ECC arrest adjusting for confounders. The interaction between the type of intervention and baseline lesion severity, moderate (ICDAS 3/4) or advanced (ICDAS 5/6), was also assessed.

**Results:**

The study included 165 children with 949 active lesions. After 6 months, there were no significant differences between SDF and NaF/MI groups in overall caries arrest (63.7% and 58.1%, *p* = 0.08), and in moderate lesions (72.9% and 69.6%, *p* = 0.52). However, in advanced lesions, the arrest rate was significantly higher in the SDF than the NaF/ MI group (60.3% and 50.0%, *P* = 0.01). Multilevel multiple logistic regression showed no significant differences between the interventions (AOR = 1.56, *P* = 0.27) with significant interaction between the intervention and baseline lesion severity (*p* < 0.001). Moderate lesions treated with SDF (AOR = 3.69, *P* = 0.008) or NaF/MI (AOR = 3.32, *P* < 0.001) had significantly higher odds of arrest than advanced lesions treated with NaF/ MI with no difference between advanced lesions treated with SDF or NaF/ MI (AOR = 1.85, *P* = 0.155) in arrest rate.

**Conclusion:**

NaF/ MI can be an alternative to SDF in arresting advanced and moderate ECC lesions without staining with stronger effect on moderate lesions (ICDAS 3/4).

**Trial registration:**

The trial was retrospectively registered at clinicaltrial.gov registry (#NCT05761041) on 9/3/2023.

## Introduction

Early childhood caries (ECC) is one of the most common chronic childhood diseases [1]. It is defined as the presence of one or more decayed, missing, or filled tooth surfaces in any primary tooth in a child under the age of six years [2]. Untreated dental caries in deciduous teeth was the 10^th^ most prevalent condition globally [3] affecting 532 million children worldwide in 2017 [4]. A global study showed that the prevalence of ECC in Egypt in children younger than 36 months and in 3–5 year-old children exceeded 50% [5]. Untreated caries leads to pain, sepsis, and eventually tooth extraction jeopardizing children’s growth and quality of life [6].

Prevention and minimally invasive dentistry [7] aim at managing carious lesions in a conservative manner to preserve the tooth structure [8]. Fluorides have been used to prevent the development of carious lesions and prevent caries progress. Sodium Fluoride (NaF) varnish is a common fluoride product for caries prevention [9, 10]. Previous studies have shown positive results in arresting enamel caries when 5% NaF varnish was used [11, 12]. However, 5% NaF varnish has low efficacy in arresting dentin caries [13]. Silver Diamine Fluoride (SDF) is another fluoride product that has gained attention as one of the minimally invasive modalities to manage ECC. SDF has an antibacterial effect, inhibits demineralization and promotes remineralization of enamel and dentin, thus arresting carious lesions [14]. However, one of the drawbacks of SDF is black staining of treated carious lesions which compromises esthetics especially in anterior teeth [15].

Health behaviors are established early in life and caregivers play a vital role in shaping their children’s health behaviors [16]. Behavior change interventions targeting oral health practices such as diet modification and regular toothbrushing may prevent ECC. Motivational Interviewing (MI) is a technique that can promote healthy behaviours [17]. It is a patient-centered, collaborative counseling approach designed to strengthen an individual’s intrinsic motivation towards a positive behavior [18]. MI uses change-talk and reflective listening to motivate patients to modify their behaviors by respecting autonomy and enabling the patient to feel engaged, understood and empowered. MI was effective to various degrees in preventing ECC in clinical trials involving pregnant women and mothers of young children [19, 20]. It also had an impact on caries severity in indigenous children [19] and on reducing the number of surfaces affected by ECC compared to conventional oral health education [20].

SDF has shown superior results to NaF in arresting caries [13, 21]. However, MI that promotes toothbrushing with fluoridated toothpaste may lead to sustained fluoride exposure in addition to reducing plaque accumulation, thus, having additional caries preventive effect when used with NaF varnish. Such a behavior-based intervention would potentially avoid the black staining caused by SDF and institute good oral health practices that promote sustainable control of caries when children are young and as they grow.

The aims of this study were to compare the effectiveness of 38% SDF versus 5% NaF varnish supported by MI sessions for mothers in arresting ECC and to assess the interaction between the type of intervention and baseline lesion severity on ECC arrest in addition to comparing parents’ satisfaction with their children’s dental appearance. The null hypothesis was that there would be no significant difference between the two interventions in arresting ECC.

## Materials and methods

This randomized, parallel, two- arm clinical trial was conducted in nurseries of rural areas around Alexandria, Egypt, from March 2022 to March 2023. Ethical approval was obtained from the Research Ethics Committee at the Faculty of Dentistry, Alexandria University, Egypt (#0272–07/2021). The research purposes and procedures, risks and benefits were explained to the parents and they were asked to sign an informed consent form. The study was conducted in accordance with the Helsinki declaration for human research [22]. The trial was registered at clinicaltrial.gov registry (#NCT05761041). Reporting of the study followed the CONSORT guidelines [23].

### Participants eligibility

The inclusion criteria were children ≤ 4 years old with at least one active carious lesion on a primary tooth with ICDAS score of 3 to 6. The activity of the lesion was assessed using the ICDAS-LAA criteria where ICDAS 3 lesions were active if the enamel surface was opaque with loss of luster and present in plaque stagnation areas as pits and fissures, near gingival margin or in proximal surfaces below contact point. ICDAS 4 lesions were all considered probably active. ICDAS 5 or 6 lesions were identified as active if softness was detected on gentle probing [24, 25]. Children were excluded if they had spontaneous or elicited pain, signs of pulpal infection, or prematurely mobile teeth. Children with history of major systemic diseases or known allergic reaction to fluoride or silver were excluded.

### Interventions

One group received 38% SDF (Advantage Arrest, Elevate Oral Care, FL, USA) and the other group received 5% NaF varnish (Alpha-Pro® White Varnish, Dental Technologies, USA) combined with 2 MI sessions, at baseline and after three months.

At the time of the intervention, teeth were cleaned and partially isolated with gauze and cotton rolls. No attempt was made to remove carious tissues before the application of materials. The isolated lesions were dried with cotton pellets.

In the SDF group, petroleum jelly was applied as a protective covering on the lips and perioral skin to avoid staining. One drop of SDF per patient was dispensed into a plastic dappen dish. A disposable microbrush was bent and dipped into SDF and dabbed on the side of the dish to remove excess liquid before applying on the affected tooth. Carious lesions were painted for 10 s and the excess was removed using cotton pellets. The solution was left to dry for one minute before the child was allowed to close their mouth. The application was done once at baseline. In the NaF/ MI group, 0.5 ml of NaF varnish was applied to the whole dentition once at baseline using a disposable brush. The entire process took 3–4 min. After applying the fluoride agents, the children were asked not to eat or drink for 30 min. Oral hygiene instructions were given to all caregivers in the two groups. After 24 h, caregivers were contacted by phone to check if there were any adverse effects.

Before providing MI, the principal investigator (RY) completed the online course “Motivational Interviewing -Interventions in Health Care” [26], and further received training by a consultant (W.H). MI sessions were provided to 3 mothers not included in the study who were attending the Pediatric Dentistry Clinic, Faculty of Dentistry, Alexandria University. We obtained their approval to record the sessions and the expert’s feedback was sought to ensure fidelity of intervention.

The mothers in the NaF/ MI group received 2 MI sessions:10–15 min face-to-face session at baseline and a 5-min phone call after 3 months for reinforcement. Fifteen mothers could not leave their homes to attend the face-to-face session after randomization and received MI over the phone at baseline. A previous study reported no significant differences between face-to-face and phone call MI [27]. The MI session started by establishing rapport and showing concern to get the mother to discuss her understanding of oral diseases, their impact on the child’s life and the child’s oral health status and oral healthcare. Open-ended questions were used and positive efforts were affirmed. This was followed by paraphrasing the oral health problems faced by the children and the mother’s goals for the child’s oral health using reflective listening and summarizing the mother’s problem and goals. The investigator exchanged information with the mother using the elicit–provide–elicit framework. The mother was asked what she knew about preventing caries progress to correct misconceptions. Also, having the mother voice this knowledge often served as change talk by implicitly stating the need for health behavior change and the consequences of not doing so. This was followed by providing information in manageable chunks, then asking again an open question to elicit the mother’s response to the information provided. Finally, after identifying the mother’s desire to change the behavior related to the child’s dental health, options and strategies for change were discussed to settle on what change she felt confident to try. The mothers were contacted after three months by phone to reinforce commitment to the new behaviour and provide support. Without follow-up, new behaviours may not be tried out or even maintained, leading to relapse.

### Outcome assessment

To prepare for clinical assessment, three examiners RY, MQ, EA received training using the 90-min ICDAS e-Learning programme by the ICDAS Foundation [28]. Examination of 10 children was conducted in the clinic of the Department of Pediatric Dentistry and Dental Public Health, Faculty of Dentistry, Alexandria University followed by re-examination after seven days to assess inter- and intra-examiner agreement measured by Cohen’s Kappa which ranged from 0.86- 0.94 indicating excellent agreement between examiners and across time [29].

The primary outcome of the study was lesion arrest after six months. Caries lesion activity was assessed using the ICDAS-LAA criteria [24, 25]. Lesions diagnosed as ICDAS code 3 or 4 at baseline were classified as arrested if after 6 months, they had not transitioned to a higher ICDAS code. A lesion that was ICDAS 5 or 6 at baseline was classified as arrested if it was hard and not leathery or soft when the probe was gently drawn over it after 6 months. Oral examination was conducted under daylighting conditions, without magnification or drying, using the World Health Organization CPI periodontal probes (405/WHO probe) with disposable dental mirrors. No radiographic examination was used.

The secondary outcome was parents’ satisfaction with their children’s dental appearance assessed by a self-rated questionnaire using a 4-point Likert scale at baseline and 6-month follow-up. The responses to the question “are you satisfied by your child’s appearance?” were very satisfied, satisfied, unsatisfied, very unsatisfied and were recoded from 1 to 4 respectively [21]. Another secondary outcome was caries increment which is the number of new caries lesions developed over the study period. This outcome is being addressed in another publication.

To control for potential confounders, we used the Arabic validated version [30] of the WHO questionnaire- child form [31] to collect information about the children’s demographic characteristics (age, sex, mother’s education) and oral-health related behaviors such as toothbrushing (at least once daily or less), dental visits during the previous year (at least once or less) and sugar consumption at least once daily or less including eight types of sugary foods/snacks: fruit, biscuits and cakes, carbonated beverages, jam and honey, sugar-added chewing gums, candies, sugar-sweetened milk and sugar-sweetened hot drinks. The sugar consumption score was created by adding the points of each of the eight products if they were consumed at least once daily. The score ranged from 0 to 8 where higher scores indicated greater daily sugar consumption [32]. Oral hygiene was assessed using the plaque index (PlI) of Silness and Loe [33] on 6 index teeth (#52, 55, 64, 72, 75, and 84) and averaging the scores (which ranged from zero, no plaque accumulation to 3, abundant visible plaque) to obtain the child’s score. The child’s caries experience was assessed using decayed, missing, and filled teeth (dmfs) index according to the World Health Organization (WHO) criteria [31].

Data were collected using an electronic form uploaded to the online platform (KoboToolbox) that allowed offline data collection with subsequent synchronization when internet access was available [34]. This method suited the rural setting where internet was not accessible all the time.

### Sample size calculation

Sample size was estimated assuming 5% alpha error and 80% study power. The caries arrest rates after 6 months using 38% SDF solution and 5% NaF varnish were 43.9% [35] and 12.3% [21]. According to Lo et al., [36] oral health education and daily toothbrushing resulted in 45% caries arrest rate in a 3-year period, with an arrest rate after 6 months estimated to be 7.5% due to brushing with fluoridated toothpaste like that induced by MI. Thus, it was assumed that the caries arrest rate after applying 5% NaF with MI would be 19.8%. Based on comparison of proportions, the required sample size per group was 58 lesions [37]. An expected drop-out rate of 40% was anticipated due to difficulty in reaching mothers in rural settings raising the required number per group to 82 lesions. Assuming an intra class correlation coefficient (ICC) = 0.3 [38] and an average number of lesions per child = 6.3 based on a pilot study conducted among a similar population, the design effect would be 2.6. The required number per group to accommodate the clustering of lesions within a child’s oral cavity = sample size* design effect [39] 82*2.6 = 213.2≈ 213 lesions per group to be multiplied by 4 for all subgroups (moderate and advanced lesions in each intervention group) with a grand total of 852 lesions or 426 lesions per intervention group.

### Randomization, allocation concealment, and blinding

Participants were randomly assigned in a 1:1 ratio using a computer-generated list of random numbers [40] in blocks of four. The allocation sequence was concealed in opaque, sealed envelopes which were arranged sequentially by a trial-independent individual. No blinding was done because MI was provided to one of the two groups and because of the staining by SDF.

### Statistical analysis

Data were analyzed using SPSS 25.0 for Windows (SPSS Inc., Chicago, USA). Significance was set at *P* < 0. 05. Intention-to-treat analysis was used and conducted at the tooth surface level. The carious lesion status of lost-to-follow-up participants was recorded using the worst-case scenario and they were considered active lesions. Also, carious lesions that received operative or surgical treatment were considered active at follow-up [21]. Chi-squared test was used to compare the groups regarding baseline demographic background, oral health behaviors, and caries arrest rate. The groups were compared regarding age, sugar consumption scores, plaque index scores, and dmfs scores using independent samples t-test or Mann–Whitney U test based on whether they were normally distributed.

A multilevel binary logistic regression analysis was used to assess the effect of the intervention on lesion activity (active versus not active) after 6 months, controlling for confounders (age, sex, mother’s education, tooth surface categorized into proximal, buccal/ lingual or occlusal, baseline dmfs score, baseline lesion severity measured by ICDAS score (classified into moderate lesions- ICDAS 3/ 4 and advanced lesions- ICDAS 5/ 6), brushing frequency, dental visits frequency, and sugar consumption score). The plaque index score was removed from the regression model due to collinearity with tooth brushing frequency. Adjusted odds ratios (AORs), 95% confidence intervals (CIs) and p values were calculated. The interaction of baseline ICDAS score (moderate and advanced lesions) and intervention was assessed by calculating the p value of the interaction followed by calculating the AORs comparing the four subgroups.

## Results

A total of 165 children with 949 active lesions participated in the study. Table 1 shows that there were no significant differences at baseline between the groups in demographic background except mother’s education (*P* = 0.002). There were no significant differences between groups in oral health behaviors including sugar consumption (*p* = 0.78), toothbrushing frequency (*p* = 0.08) and dental visits last year (*p* = 0.10). There were no significant differences in the dmfs score and plaque index (*p* = 0.14 and 0.07). There were, however, significant differences in baseline severity (*p* < 0.0001) with a greater percentage of more advanced lesions in the SDF than the NaF/ MI groups. There was also a significant difference (*p* < 0.001) in included teeth surfaces with a greater percentage of occlusal surfaces in the NaF/ MI than the SDF group.

**Table 1:** Demographic background, oral health-related behaviours, and clinical characteristics of participants at baseline

		SDF group *N* = 82	NaF/ MI group *N* = 83	*P* value
**Socio-demographic background**				
Child age in months	Mean (SD) [Min, max]	42.93 (7.52) [17, 48]	43.73 (8.10) [15, 48]	0.51
Child sex	Male	38 (46.34%)	39 (46.99%)	0.93
	Female	44 (53.66%)	44 (53.01%)	
Mother’s educational level	Less than high school	36 (43.90%)	56 (67.47%)	0.002*
	High school and higher	46 (56.10%)	27 (32.53%)	
**Oral health behaviors**				
Sugar score	Mean (SD)	6.67 (1.25)	6.72 (1.09)	0.78
Tooth brushing once or more daily	Yes	13 (15.85%)	6 (7.23%)	0.08
	No	69 (84.15%)	77 (92.77%)	
Dental visits last year	Yes	46 (56.10%)	36 (43.37%)	0.10
	No	36 (43.90%)	47 (56.62%)	
**Clinical characteristics**				
dmfs	Mean (SD)	10.71 (6.90)	8.80 (9.47)	0.14
Plaque Index	Mean (SD)	1.84 (0.30)	1.94 (0.36)	0.07
Baseline caries severity^a^	ICDAS 3 or 4	133 (27.25%)	191 (41.43%)	< 0.001*
	ICDAS 5 or 6	355 (72.75%)	270 (58.57%)	
Tooth Surface^a^	Buccal/ Lingual	138 (28.28%)	104 (22.56%)	< 0.001*
	Occlusal	130 (26.64%)	196 (42.52%)	
	Proximal	220 (45.08%)	161 (34.92%)	

^*^Statistically significant at *P* < 0.05

^a^Lesions numbers shown (*n* = 488 in SDF group and 461 in NaF/MI group). All other numbers are children

**Fig. 1:**
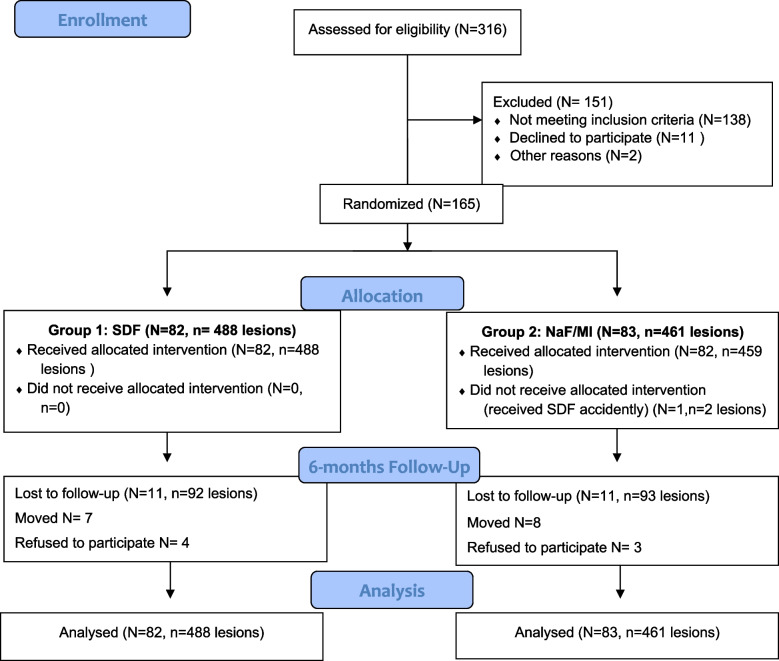
Flow chart showing flow of participants in the study

In the SDF group, there were 82 participants with 488 carious lesions and in the NaF/ MI group, there were 83 participants with 461 carious lesions at baseline. After 6 months, 22 participants were lost to follow up; 13.4% in the SDF group and 13.3% in the NaF/ MI group (Fig. 1). One child in the NaF/MI group accidentally received SDF and was analyzed with the NaF/ MI group following the intention to treat approach. No adverse events were reported by caregivers during the study period.

Table 2 shows that there were no significant differences between the SDF and NaF/ MI groups in overall ECC arrest rate (*P* = 0.08) and in moderate lesions (*P* = 0.52). However, in advanced lesions, the arrest rate was significantly higher in the SDF than the NaF/ MI group (60.3% and 50.0%, *P* = 0.01).

**Table 2:** Caries arrest rates of active caries lesions in the study groups after 6 months in bivariate analysis, overall and by baseline lesion severity

	SDF group n/N (%)	NaF/ MI group n/N (%)	*P* value
All lesions	311/488 (63.7)	268/461 (58.1)	0.08
Moderate (ICDAS 3 or 4) lesions	97/133 (72.9)	133/191 (69.6)	0.52
Advanced (ICDAS 5 or 6) lesions	214/355 (60.3)	135/270 (50.0)	0.01*

n: number of arrested lesions

N: number of included lesions

^*^Statistically significant at *P* < 0.05

Table 3 shows the results of the multi-level multivariable regression analysis. Active lesions treated with SDF were 1.56 times more likely to be arrested after 6 months than lesions treated by NaF/MI although the difference was not statistically significant (95%CI: 0.71, 3.42, *P* = 0.27) with more (76.8%) lesions in the SDF group arrested than in the NaF/ MI group (68.0%).

**Table 3:** Multivariable multilevel logistic regression model for factors affecting ECC arrest after 6 months

Explanatory variables		AOR (95% CI)	*P* value
**Sociodemographic background**			
Child age in months		1.02 (0.97, 1.08)	0.35
Child sex	Male	0.97 (0.46, 2.07)	0.94
	Female^a^	1.00	-
Mother’s educational level	Less than high school	1.16 (0.52, 2.57)	0.72
	High school and higher^a^	1.00	-
**Oral health behaviors**			
Sugar score		0.76 (0.54, 1.06)	0.10
Toothbrushing once or more daily	Yes	0.94 (0.29, 3.05)	0.92
	No^a^	1.00	-
Dental visits last year	Yes	0.95 (0.43, 2.08)	0.89
	No^a^	1.00	-
**Clinical characteristics**			
Baseline dmfs		1.00 (0.95, 1.05)	0.89
Tooth Surface	Buccal/Lingual	1.47 (0.86, 2.51)	0.16
	Occlusal	0.70 (0.43, 1.13)	0.14
	Proximal^a^	1.00	-
Baseline caries severity	Moderate (ICDAS 3/4) lesions	2.64 (1.64, 4.23)	< 0.001
	Advanced (ICDAS 5/6) lesions^a^	1.00	-
Arm	SDF	1.56 (0.71, 3.42)	0.27
	NaF/MI^a^	1.00	-

*AOR* adjusted odds ratio, *CI* confidence interval, ^a^reference category

There were no differences in arrest rates by child’s age (*p* = 0.35), child’s sex (*p* = 0.94), mother’s educational level (*p* = 0.72), sugar score (*p* = 0.10), brushing frequency (*p* = 0.92), dental visits last year (*p* = 0.89), baseline dmfs (*p* = 0.89), or tooth surface (p > 0.05). Moderate lesions had significantly higher odds of becoming arrested than advanced lesions (AOR: 2.64, 95%CI: 1.64, 4.23, *P* < 0.001).

The interaction between the intervention and baseline lesion severity was significant (*p* < 0.001). Moderate lesions treated with SDF (AOR = 3.69, 95% CI:1.40, 9.70) or NaF/MI (AOR = 3.32, 95%CI: 1.76, 6.25) had significantly higher odds of arrest than advanced lesions treated with NaF/ MI. There was no difference in the arrest between advanced lesions treated with SDF or NaF/ MI (AOR = 1.85, 95% CI: 0.79, 4.31). The percentages of arrested lesions were higher in moderate than advanced lesions and in SDF-treated than NaF/MI treated lesions with greater difference between agents in advanced than moderate lesions (Table 4).

**Table 4:** Interaction between baseline lesion severity and intervention

Group	Percent arrested lesions (95%CI)	AOR (95%CI)	*P* value
SDF x ICDAS 3/4	81.5 (65.7, 91.0)	3.69 (1.40, 9.70)	0.008
NaF/MI x ICDAS 3/4	79.9 (62.6, 90.4)	3.32 (1.76, 6.25)	< 0.001
SDF x ICDAS 5/6	68.8 (52.2, 81.7)	1.85 (0.79, 4.31)	0.155
NaF/MI x ICDAS 5/6^a^	54.5 (34.6, 73.0)	1.00	-

Adjusted for sex, age, mother’s education, sugar consumption, dental visits, tooth brushing frequency, tooth surface, and baseline dmfs

*AOR* adjusted odds ratio, *CI* confidence interval, ^a^reference category

Table 5 shows that 86.6% and 89.2% of mothers were satisfied with their children’s dental appearance after the application of SDF and NaF/ MI, respectively. After 6 months, the percentages of satisfied parents decreased to 76.1% and 84.5% respectively. There were no significant differences between groups at baseline or after 6 months (*P* = 0.61 and 0.21). The changes in the percentage of satisfied parents from baseline to 6 months in the SDF group (*P* = 0.19) and the NaF/ MI group (*P* = 0.23) were not statistically significant.

**Table 5:** Parental satisfaction with the children’s dental appearance at baseline and after 6 months

	SDF group n/N (%)	NaF/ MI group n/N (%)	P of X2 test
Baseline			
Satisfied	71/82 (86.6)	74/83 (89.2)	0.61
Unsatisfied	11/82 (13.4)	9/83 (10.8)	
6-month			
Satisfied	54/71 (76.1)	60/71 (84.5)	0.21
Unsatisfied	17/71 (23.9)	11/71 (15.5)	
P of McNemar test	0.19	0.23	

n: number of participants with specific response

N: total number of participants

## Discussion

In 15–48-month-old Egyptian children living in rural areas, ECC arrest rates after 6 months did not differ significantly when one application of 38% SDF or 5% NaF combined with 2 MI sessions were used. Thus, the null hypothesis of the study was not rejected. There was a significant interaction between the intervention and baseline lesion severity with stronger effect of SDF than NaF/ MI in advanced than in moderate lesions. The study fills a knowledge gap by providing evidence on the effects of a potent caries arrest agent, SDF, and the traditional NaF varnish combined with MI targeting mothers’ behavior.

The present study has some limitations. ECC was assessed clinically using visual-tactile examination without radiographs. Although this has the potential to miss some caries lesions, the outcome in the study was caries arrest which was assessed using visual-tactile sensation following the reported criteria. The same method was also used in previous studies assessing the effectiveness of fluoride agents [11, 21]). In addition, there is a potential for some detection bias because SDF-treated lesions were stained black and, thus, may have been more likely to be recorded as arrested than lesions in the other group. Also, the study duration was relatively short. However, the literature shows that caries arrest can be assessed after similar [41] or shorter periods [42, 43]. In addition, sample size was based on arrest rates difference between groups of 24% whereas the difference in the study proved to be much less (< 6%). This might have affected the study power and shows the importance of conducting studies to generate context-specific estimates for the different caries preventive agents. ECC is a complex disease that is affected by individual, familial and community factors based on biologic, microbial, dietary, and cultural determinants making it unrealistic to expect the same caries preventive effect in two settings that differ widely in underlying determinants. Further studies are needed to assess the long-term effects of NaF/MI on ECC arrest and to investigate whether no difference in arrest rates would remain after longer periods. On the other hand, the study has several strengths including the rural setting where children are in greatest need of minimally invasive modalities to arrest caries and subvert extensive and costly dental care. Our study also ensured adequate power by including a large number of carious lesions. These lesions were the unit of analysis and because of this, we used multilevel analysis that can accommodate the clustering of lesions within children.

In the present study, there was no difference in ECC arrest rate between SDF and NaF/ MI in multivariable analysis. SDF had a significantly higher caries arrest rate in advanced lesions although this significance disappeared after adjusting for confounders. By contrast, SDF has been previously reported to be highly effective in arresting dentin carious lesions, [44] with greater arrest rate of dentin caries than NaF [13]. The difference between our study and the previous studies may be attributed to MI which induced behavior change and improving oral hygiene. Dental plaque is the driving force for demineralization and caries progression [45]. Thus, reducing plaque accumulation and decreasing the bacterial load is key to caries arrest. A previous study showed that 45% of anterior carious lesions were arrested after 36 months due to a toothbrushing intervention [36]. We propose that good oral hygiene following MI complemented NaF to better control ECC thus reducing the relative advantage of SDF that was reported in previous studies.

The study findings also show that there was a non-significant difference between SDF and NaF/ MI in arresting moderate lesions. This agrees with Duangthip et al. [11] who reported a small and non-significant difference between 30% SDF and 5% NaF in arresting ICDAS 3 or 4 lesions. Our findings disagree with Phonghanyudh et al. [12] who reported that 38% SDF had significantly higher arrest rate of ICDAS 3 enamel lesions than NaF after 6 months. However, Phonghanyudh et al. applied NaF varnish on affected lesions only unlike the present study where NaF varnish was applied to all teeth with greater area available to act as fluoride reservoir and continuously release fluoride than when applied on affected lesions only [46].

There is scarce evidence on the effectiveness of SDF versus NaF supported by MI in arresting ECC. MI was reported to improve the oral health knowledge and attitudes of caregivers leading to improved oral health behaviors [47, 48]. Also, lower caries incidence was reported in children whose parents received MI [20, 49, 50]. One study attributed the observed caries prevention to more frequent visits to the dentist by children whose parents received MI which allowed children to receive fluoride varnish treatment [49].

Although black staining is a drawback of SDF, there was no significant difference in parental satisfaction between groups receiving SDF and those with NaF/ MI in the present study. It seems that the caregivers’ main concern was to stop caries progression regardless of the esthetics. These findings agree with previous studies [12, 15, 21].

It is important to interpret the findings within the context of the study setting. Children in previous studies assessing the effect of SDF and NaF varnish on arresting caries had higher tooth brushing frequency [12, 21]. In the present study, however, the children had high levels of plaque accumulation and low tooth brushing frequency which highlight the need for behavior change interventions to promote good oral hygiene and control ECC. If caries promoting factors, such as poor oral hygiene, are prevalent, the disease progresses and new lesions keep developing. SDF application should not be regarded as a magic solution to control ECC under these circumstances. Addressing the behaviors that increase ECC risk is important to stop ECC progress and provide a sustainable, patient centered solution to the problem. However, NaF application combined with MI face-to-face session take at least 10–15 min in addition to 5 more minutes for a reminder phone call. This intervention may, thus, be more time consuming than SDF application which requires only 3–4 min. Cost is another factor that should be considered and requires further research.

The current findings show that NaF/ MI can be an alternative to SDF for arresting ECC without staining the treated lesions. Behavior modification using MI combined with traditional NaF varnish may adequately arrest ECC in children with modest oral hygiene without the need to use SDF.

## Conclusion

Based on the 6-month results of the current study, there was no significant difference between SDF and NaF/MI in arresting carious lesions. NaF/ MI can be an alternative to SDF to arrest ECC especially moderate lesions (ICDAS 3/4) where the difference between the two regimens was minimal. Studies with longer follow up periods are needed to assess the long-term effects of NaF/MI on ECC arrest.

## Data Availability

The data used and analyzed in the present study are available from the corresponding author on reasonable request.

## Abbreviations

ECC: Early Childhood Caries
MI: Motivational Interviewing
SDF: Silver Diamine Fluoride
NaF: Sodium Fluoride
ICDAS: International Caries Detection and Assessment System
ICDAS-LAA: International Caries Detection and Assessment System—Lesion Activity Assessment
PI: Plaque index
SPSS: Statistical Package for Social Sciences
